# Transarticular fixation by hook plate versus coracoclavicular stabilization by single multistrand titanium cable for acute Rockwood grade-V acromioclavicular joint dislocation: a case–control study

**DOI:** 10.1186/s12891-015-0820-y

**Published:** 2015-11-19

**Authors:** You-Shui Gao, Yue-Lei Zhang, Zi-Sheng Ai, Yu-Qiang Sun, Chang-Qing Zhang, Wei Zhang

**Affiliations:** Department of Orthopedic Surgery, Shanghai Jiao Tong University Affiliated Sixth People’s Hospital, Shanghai, 200233 China; Department of Medical Statistics, Tongji University School of Medicine, Shanghai, 200092 China

**Keywords:** Acute acromioclavicular dislocation, Hook plate, Multistrand titanium cable, Coracoclavicular stabilization, Young adults, Rockwood classification

## Abstract

**Background:**

Hook plate (HP) is popularly used for acute and severely displaced acromioclavicular (AC) dislocations. However, subacromial impingement and acromion osteolysis induced by transarticular fixation are notorious. The current case–control study was to compare transarticular fixation by HP to coracoclavicular (CC) stabilization by single multistrand titanium cable (MSTC).

**Methods:**

Between January 2006 and August 2009, 24 patients with acute AC dislocations were surgically treated by open reduction and transarticular fixation with HP. These patients were matched to a series of 24 patients, who were managed by CC stabilization with MSTC in the same period. All AC dislocations were graded as Rockwood type V. Implant was removed 8 -- 12 months after the primary operation in all patients, and 12 months at least were needed to assess the maintenance of AC joint. Functional results were evaluated before implant removal as well as in the last follow-up based on Constant-Murley criteria.

**Results:**

There were no differences of demographic data including age, dominant gender and side, injury-to-surgery interval, operation time and follow-up period. In terms of functionality, Constant score was 95.8 ± 4.1 in MSTC group, while 76.7 ± 8.0 in HP group before implant removal (*P* < 0.001). In detail, MSTC was superior to HP in pain, ROM and activities. Constant score was significantly improved to 86.1 ± 5.7 after hardware removal for patients in HP (*P* < 0.001). Degenerative change of acromioclavicular joint presented in 16 patients (66.7 %) in patients treated by HP, while it was found in only 3 patients (12.5 %) treated by MSTC (*P* < 0.001).

**Conclusions:**

MSTC is superior to HP for the treatment of Rockwood type-V acromioclavicular dislocation both before and after removal of the implant. Hardware removal is of great benefits for functional improvement in patients treated by HP.

## Background

Acromioclavicular (AC) joint dislocation is a common injury in active young adults. AC dislocation is associated with AC and coracoclavicular (CC) ligaments injuries and different levels of distal clavicle dislocation, which are combined for the judgment of grades of AC dislocation according to Rockwood classification [1, 2]. Conventionally, grades I to III AC dislocations could be treated by conservative treatment [3], while higher grades of injuries should be treated surgically [4, 5].

A plethora of surgical methods have been employed for the treatment of AC dislocations including transarticular fixation by hook plate (HP) and Kirschner wires, as well as extraarticular fixation by CC restoration with metallic cables, autologous ligament or LARS artificial ligament [6–14]. According to a recent survey in Germany, HP and TightRope techniques are preferable [15]. Transarticular fixation with HP might induce bony erosion, shoulder impingement and rotator cuff damage, which can result in poor functional results [16, 17]. However, extraarticular fixation is not perfect as expected [18, 19]. In past five years, there are varieties of studies to compare different methods for the treatment of acute and chronic AC dislocations [6, 16, 20]. Eschler and colleagues found HP fixation restored the CC distance more accurately than augmentation with a PDS sling [16], while Takase and Yamamoto indicated anatomic restoration of both AC ligaments could best restore AC joint function [6]. The consensus is difficult to be achieved due to the lack of case–control or prospective randomized studies.

The current retrospective case–control study is designed to compare transarticular fixation by HP and CC fixation by multistrand titanium cable (MSTC) for grade-V AC dislocation. The hypothesis includes two aspects:

1. MSTC is a superior fixation technique for Grade-V AC dislocation in comparison with HP; and
2. hardware removal is beneficial after a follow-up period of 8 ~ 12 months.

To our knowledge, this is the first case–control study to compare trans- and extra-articular fixation with different methods for grade-V AC dislocation.

## Methods

### Patients

The ethics committee of Shanghai Jiao Tong University Affiliated Sixth People’s Hospital approved the current study, and patients provided informed consent prior to our study. All patients agreed that all medical data, including their personal and radiographic photographs, could be sent for academic publication. Between January 2006 and August 2009, 24 patients with acute AC dislocations were surgically treated by open reduction and transarticular fixation with HP. These patients were compared to a series of 24 patients, who were managed by CC stabilization with MSTC in the same period. All AC dislocations were graded as Rockwood type V, namely, all stabilizing anatomical structure including the CC and AC ligaments and delto-trapezoidal fascia were disrupted and the CC interval was widened between 100 and 300 % with substantial widening of the AC distance [1, 2]. Transarticular fixation with HP and CC fixation with a single MSTC are both commonly used methods for surgical treatment of acute AC dislocations in authors’ institute. There was no inclusion bias for two different surgical methods.

Indications for surgical treatment included: acute Rockwood grade-V AC dislocation, young adults (20–50 years), no history of bone diseases or associated metabolic diseases and no history of concomitant shoulder diseases. There was no other concomitant skeletal trauma except the confirmed AC injury. Preoperative MRI was not routinely used [21]. Patients enrolled in the current study were these had operative indications, and implant removal 8 ~ 12 months postoperatively, and 12 months further at least to assess the maintenance of AC joint as well as final functionality. The demographic data in detail were listed in Table 1.

**Table 1:** Demographic data in two different groups

		HP	MSTC	*P* value
Age (years)		36.0 ± 6.7	35.4 ± 8.6	0.795^b^
Gender (M:F)		19:5	18:6	0.731^a^
Side (R:L)		17:7	16:8	0.756^a^
Injury-to-surgery interval (days)		2.6 ± 1.6	2.8 ± 1.5	0.644^b^
Operation time (minutes)		37.9 ± 6.7	40.8 ± 5.6	0.12^b^
Follow-up (months)	before implant removal	10.7 ± 0.9	11.1 ± 0.9	0.08^b^
	after implant removal	18.3 ± 8.0	18.8 ± 7.5	0.825^b^

^a^ *χ* ^2^ test with continuity correction; ^b^Student *t* test

### Surgical methods

**Fig. 1:**
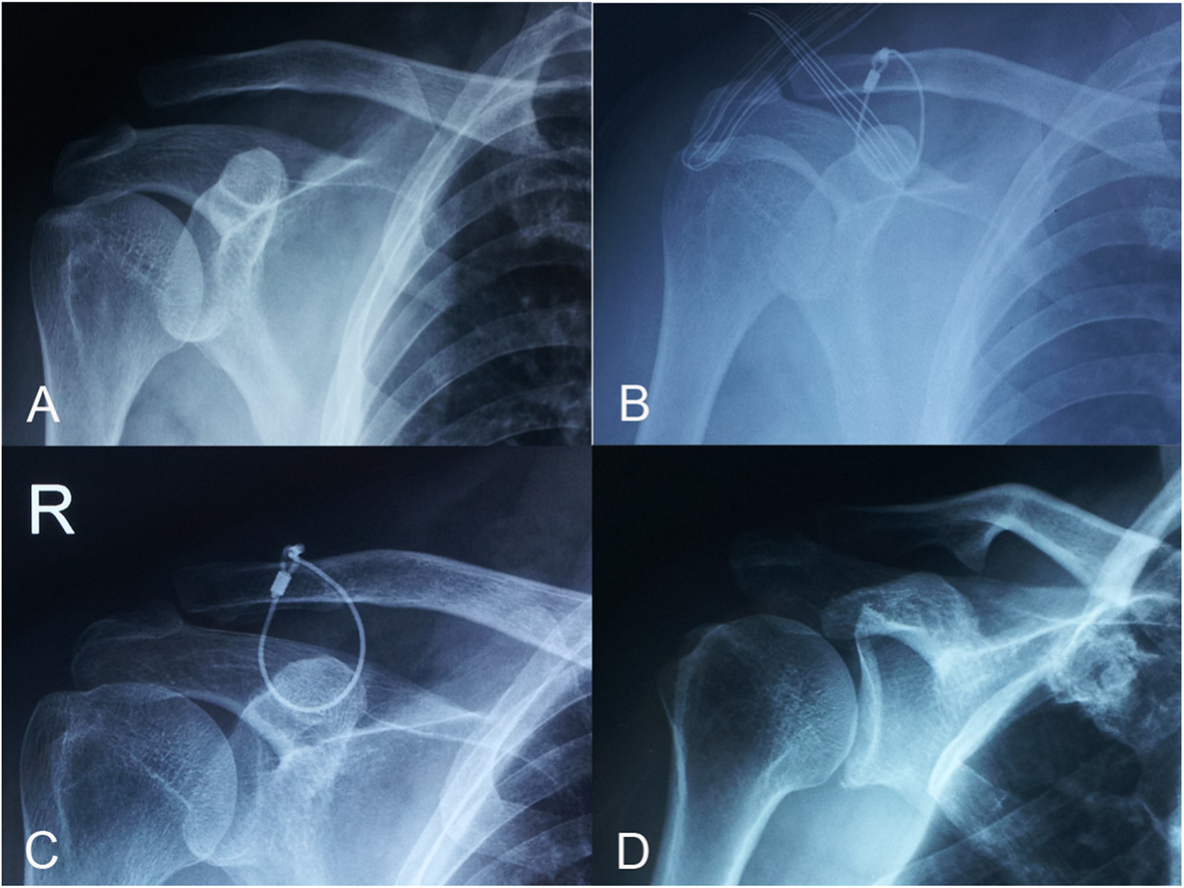
Acute AC joint dislocation treated by multistrand titanic cable (MSTC). **a** Anteroposterior view of the shoulder showed Rockwood type-V AC joint dislocation. **b** AC joint dislocation was managed by open reduction and internal fixation by MSTC in a figure-of-eight fashion. **c** Slight loss of reduction was found 3 months postoperatively. **d** The position was maintained after hardware removal

The patient was placed in a beach-chair position under general anesthesia or nerve block of brachial plexus. When HP stabilization was the treatment of choice, a longitudinal incision along the lateral clavicle was used to expose the AC joint. The dislocated lateral clavicle was reduced and temporarily stabilized by transarticular Kirschner wires from lateral acromion. After satisfactory reduction was achieved, the hook was inserted along the posterior edge of the AC joint, and the plate above the clavicle was screwed. Anterior capsule of the AC joint as well as deltotrapezoid fascia was repaired when achievable. Passive motion of the glenohumeral joint was employed to detect any abnormal impingement intraoperatively. Alternatively, a perpendicular and curved incision centered the coracoid was used when MSTC was adopted. The coracoid could be palpated and easily exposed without muscular detachment. Afterwards, AC joint could be repositioned with pointed reduction clamp, and temporarily stabilized by transarticular Kirschner wires. A single multistrand titanium cable was used passing inferior base of coracoid process and locked to the lateral clavicle in a figure-of-eight style (Fig. 1). We did not drill a tunnel through the clavicle to reduce the risk of iatrogenic fractures. According to our observation, the site of MSTC on the clavicle was relatively constant, between the torn stumps of CC ligaments. The implant of hook plate was provided by Synthes (Davos, Switzerland) while MSTC was purchased from Guci (Zhejiang, China).

### Postoperative management and Clinical Evaluation

The arm was immobilized in a sling for 4 weeks, and passive motion of the shoulder was commenced thereafter. For patients treated by hook plate, passive functional exercises could be begun a little earlier. Time to active full range of motion of the shoulder depended on progress and symptoms following passive exercises.

All patients were required to conform to regular follow-up 1, 3 and 6 months postoperatively. Routine anteroposterior and lateral imaging to the shoulder was obtained to evaluate the maintenance of the distance of AC joint and the implant. Meanwhile, special attention should be paid on degenerative change of AC joint.

Constant-Murley criteria, including four items of pain, ROM (range of motion), strength and ADL (activities of daily living, mainly including sleep, work, recreation/sport), were used to evaluate functional results before hardware removal [22]. Eight to 12 months postoperatively, hardware removal was carried out, and the functionality was reevaluated when patients got recovered as well as radiograph was repeated to evaluate the AC joint.

### Statistics

All numerical data were expressed as mean ± standard deviation (SD). Student *t* test was used compare numeral data between the two groups, while *χ*^2^ test was used for non-numerical data. Student *t* test was used to compare Constant score before and after hardware removal. Statistical analysis was processed with the software SPSS 20.0, and a *P* value of less than 0.05 was considered significant.

## Results

### General results

There were 19 male and 5 female in HP group with an age of 36.0 years on average, while 18 male and 6 female in MSTC group with average age of 35.4 years (Table 1). The predominant side was right, which was involved in 17 and 16 cases in HP and MSTC group, respectively (*P* = 0.756). Injury-to-surgery interval (ISI) was 2.6 days in HP and 2.8 days in MSTC (*P* = 0.644). The operation time was 37.9 and 40.8 min in HP and MSTC group, respectively (*P* = 0.12). Follow-up period was 10.7 and 11.1 months before hardware removal (*P* = 0.08), and 18.3 and 18.8 months after that (*P* = 0.825) in HP and MSTC group, respectively.

### Functional results

Before the implant removal, the Constant score was 76.7 ± 8.0 and 95.8 ± 4.1 in HP and MSTC group, respectively. It was considered that functional results were significantly better in MSTC group (*P* < 0.001). In detail, functional results including postoperative pain, ROM and ADL in MSTC were superior to these in HP (Table 2).

**Table 2:** Functional results before implant removal expressed by Constant-Murley criteria

	HP	MSTC	*P* value^a^
Pain	9.8 ± 2.8	13.8 ± 2.2	<0.001
Strength	23.8 ± 2.2	24.6 ± 1.4	0.127
ROM	28.6 ± 6.1	38.5 ± 2.4	<0.001
ADL	14.6 ± 1.9	18.9 ± 1.3	<0.001
Constant score	76.7 ± 8.0	95.8 ± 4.1	<0.001

^a^Student *t* test

**Fig. 2:**
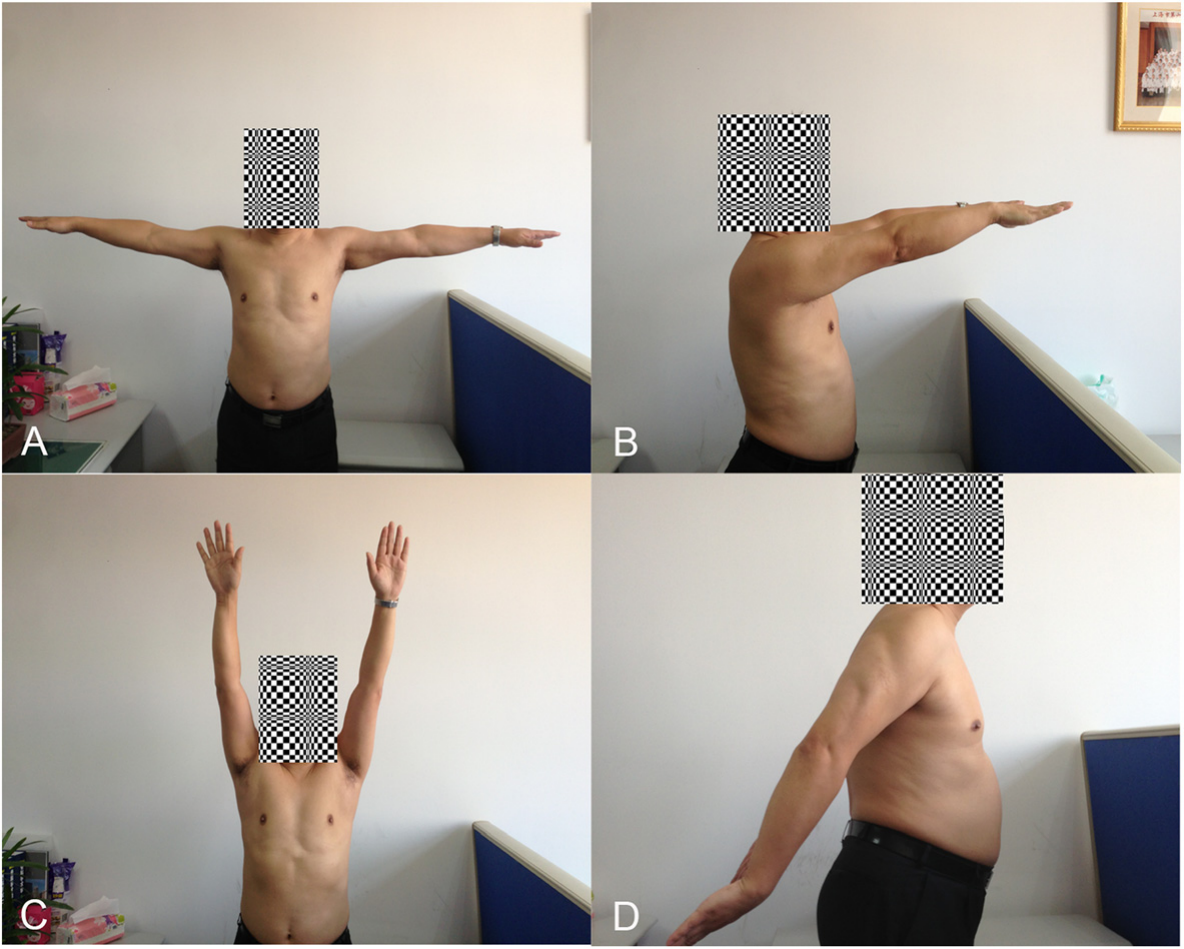
Functional results of the shoulder joint. The patient with radiographic data showed in Fig. 1 had excellent functional outcome. The involved shoulder joint had the same range of motion as the contralateral in abduction (**a**), anterior extension (**b**), elevation (**c**) and posterior extension (**d**). (The patient has provided consent to appear in the Figure)

The functional results were reevaluated after implant removal. In HP group, pain, ROM and ADL were all improved with the follow-up of 18.3 months on average (*P* < 0.001, < 0.001, = 0.002, respectively). The Constant score increased to 86.1 ± 5.7 postoperatively (*P* < 0.001). In MSTC group, it seemed items of pain, strength, and ADL were not significantly improved after implant removal (Fig. 2). However, postoperative elevation of the Constant score to 97.5 ± 2.7 was statistically better (*P* = 0.001). Therefore, it can be speculated that implant removal is beneficial for functional improvement for acute AC joint dislocation treated by HP and MSTC. Removal of hook plate is strongly recommended for its evident negative effect on functional results (Table 3).

**Table 3:** Constant-Murley score before and after implant removal

		Before implant removal	After implant removal	*P* value^a^
HP	Pain	9.8 ± 2.8	12.3 ± 2.5	<0.001
	Strength	23.8 ± 2.2	24.6 ± 1.4	0.043
	ROM	28.6 ± 6.1	33.2 ± 4.0	<0.001
	ADL	14.6 ± 1.9	16.1 ± 1.9	0.002
	Constant score	76.7 ± 8.0	86.1 ± 5.7	<0.001
MSTC	Pain	13.8 ± 2.2	14.4 ± 1.7	0.083
	Strength	24.6 ± 1.4	25.0	0.162
	ROM	38.5 ± 2.4	39.1 ± 1.6	0.032
	ADL	19.0 ± 1.3	19.1 ± 1.0	0.162
	Constant score	95.8 ± 4.1	97.5 ± 2.7	0.001

^a^Student *t* test

In comparison of final results after hardware removal between HP and MSTC group, it was revealed items of pain, ROM and ADL were all statistically better in patients treated by MSTC. Naturally, the overall Constant score was statistically higher in MSTC group (Table 4).

**Table 4:** Functional results after implant removal expressed by Constant-Murley criteria

	HP	MSTC	*P* value^a^
Pain	12.3 ± 2.5	14.4 ± 1.7	0.002
Strength	24.6 ± 1.4	25.0	0.155
ROM	33.2 ± 4.0	39.1 ± 1.6	<0.001
ADL	16.1 ± 1.9	19.1 ± 1.0	<0.001
Constant score	86.1 ± 5.7	97.5 ± 2.7	<0.001

^a^Student *t* test

### Radiographic results and complications

Postoperative infection and hardware failure did not present. There were no fractures of acromion, coracoid process or lateral clavicle. Before the implant removal, pain and restricted motion of the shoulder were common in patients treated by hook plate.

**Fig. 3:**
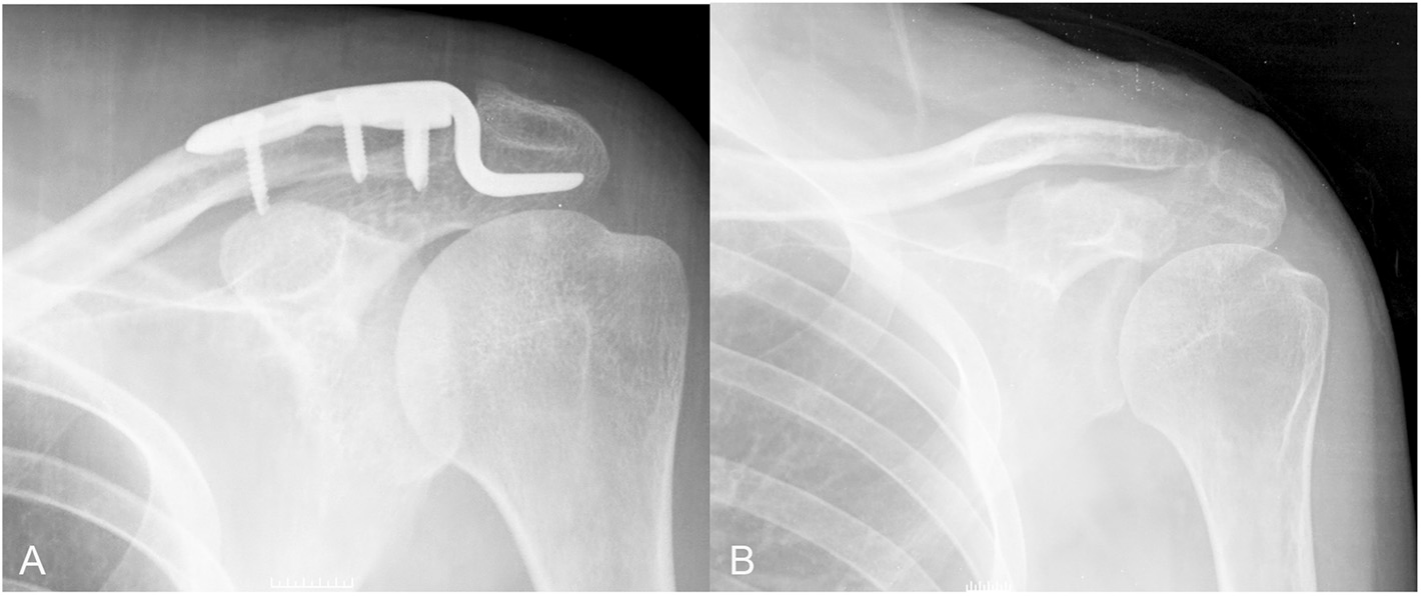
AC joint dislocation treated by hook plate resulting in degenerative osteoarthritis. **a** Anteroposterior view of the shoulder showed AC dislocation treated by hook plate. **b** Degenerative change of AC joint was presented after hardware removal

Degenerative change of acromioclavicular joint presented in 16 patients (66.7 %) in patients treated by HP (Fig. 3), while it was found in only 3 patients (12.5 %) treated by MSTC (*P* < 0.001). Degenerative change of AC joint could cause permanent pain following HP removal [23]. Before the implant removal, 3 patients in MSTC group had mild loss of reduction of AC joint. However, the loss of reduction did not progress after implant removal. There was no loss of reduction in HP group, due to its rigid characteristics.

## Discussion

Acromioclavicular joint dislocation is commonly seen in young and active adults. Various treatment modalities, including trans- and extra-articular stabilization methods, have been proposed for acute and chronic AC joint dislocation, with respective clinical results and complications [19, 24]. According to a survey made in Germany, hook plate and arthroscopy-assisted TightRope technique are the popular treatment of choice [15]. However, the majority of orthopedic trauma surgeons do not prefer arthroscope-assisted surgery, for the lack of experience in the specialist. Hook plate is also very popularly used in China. The technique is quite simple and the outcome is effective. However, transarticular fixation with HP can induce bony erosion, shoulder impingement and rotator cuff damage, which can result in unsatisfactory functional results [17]. Moreover, removal of HP is recommended as a secondary surgical intervention. The place of transarticular stabilization with hook plate is challenged by extraarticular anatomical or functional CC reconstruction. In the past decade, materials to reconstruct CC joint evolve significantly, including autologous ligament, LARS, absorbable PDS sling, screw and multistrand titanium cable. It is difficult to define an overwhelming material, because of the difference of surgical methods and lack of persuasive randomized controlled study. Orthopedic trauma surgeons are familiar to MSTC, which is usually used in tension band technique for patellar and olecranon fractures. MSTC conceives of better mechanical property than conventional cable and steel wire. In a recent study conducted by Ye and colleagues, double MSTCs were used to pass through two holes drilled in clavicle, to stabilize the CC joint. The Constant scores were 95.3 on average at final evaluation [25]. Nevertheless, risk of iatrogenic fracture was increased due to two neighboring holes in the mid clavicle. Chen and colleagues described their technique, using a tape to pass through the inferior base of the coracoid process and the clavicular tunnel in a figure-of-8 fashion [18]. The optimal position of the clavicular tunnel is difficult to be determined, due to CC ligament is not a point-like structure.

In the current study, we made a comparative study between two widely used surgical techniques for acute AC joint dislocation, transarticular stabilization with HP and extraarticular stabilization with a single MSTC. Although we believe single MSTC technique might be superior to HP according to our experience, there is no persuasive case–control study previously. As expected, extraarticular stabilization with MSTC yields better functional results, especially in sub-terms of pain, ROM and ADL, revealed by the Constant Scoring system. In detail, we found ranges of motion, including posterior extension, abduction and internal rotation, were significantly worse in patients with AC dislocation treated by hook plate, when compared to those treated by suture loop. For anterior extension, adduction and external rotation, the superiority of MSTC was not statistically significant.

Known shortcomings induced by HP include bony erosion, shoulder impingement and rotator cuff damage, which can lead to persisted pain and restricted ROM in combination or singly. Moreover, rigid and static stabilization of AC joint by HP does not reproduce the primary dynamic unit of lateral clavicle, which contributes significantly to the free motion of shoulder. Degenerative change of AC joint is persistent complication even following removal of HP. In contrast, CC stabilization by MSTC is elastic, although there are no data to show micromotion of lateral clavicle currently. We do not make osseous tunnel for MSTC for two reasons, one is to reduce risk of iatrogenic clavicle fracture, and the other is to improve the position of MSTC over clavicle. During MSTC tightened, it can slide over the clavicle and cease at an optimal position. MSTC is then locked following evaluation of AC joint reduction. In this way, the step to determine the position of clavicular tunnel could be skipped. However, it should be noted that MSTC suture loop is not flawless and perfect. Jerosch et al. once reported that suture loop could lead to anterior displacement of the distal clavicle in relation to the scapula in a biomechanical cadaveric study [26]. Increased stress by MSTC can induce bone resorption, however, it is not significant in our patients probably due to good bone quality and short time of ligamentous healing.

The limitation of current study is the number of patients is not large enough, although we believe a case–control study might be persuasive. The axillary view, which is helpful to determine anterior or posterior dislocation of the lateral clavicle, is not routinely adopted in our study. One-year follow-up after implant removal is a short time to evaluate clinical and radiological results. Moreover, maintenance of hook plate is a little too long in the current study, which might deteriorate the degenerative changes of AC joint. Additionally, the current study is retrospective, but not in a prospective and randomized fashion.

## Conclusions

MSTC is superior to HP for the treatment of Rockwood type-V acromioclavicular dislocation both before and after the hardware removal. Hardware removal is of great benefits for functional improvement in patients treated by hook plate.

## Abbreviations

HP: Hook plate
MSTC: Multistrand titanium cable
CC: Coracoclavicular
AC: Acromioclavicular
